# Supplementary material for: Multicenter automatic detection of invasive carcinoma on breast whole slide images
Source: PLOS Digit Health. 2023 Feb 28;2(2):e0000091. doi: 10.1371/journal.pdig.0000091 (PMC9974110; doi:10.1371/journal.pdig.0000091)
Supplement: S1 Text — (DOCX) [file pdig.0000091.s001.docx]

**Annex 1. Filtering process**

As described in section Methods, a filtering process is applied to the WSI with a two-fold objective: reduce the analysis to epithelial regions and discard patches that have no interest for further analysis (artifacts, no tissue, no nuclei). This allows dramatically reducing the number of patches fed to the invasive cancer classifier.

The filtering process is made of the following steps:

- epithelial regions segmentation
- discard patches that have no interest for further analysis
  - patches do not contain nuclei
  - blurry patches
  - patches with little tissue inside

The epithelial segmentation process is described in Fig A, the tissue is first segmented at zoom x1 through a simple two class otsu [1] thresholding. The resulting mask is then parsed into 256*256px tissue patches at zoom x2.5. Epithelial regions appear as dark heterogeneous regions (see Fig A). Tissue patches undergo a gaussian smoothing so as to make epithelial regions more uniform and a final two class Otsu thresholding is applied to discriminate epithelium from stroma.

Epithelial tissue is then parsed at zoom x20 into 256*256px patches which go through a final discard process, each filter is described in Table A.

**Fig A. Epithelial regions segmentation.**

This figure illustrates the WSI epithelium segmentation process. The tissue is first segmented at zoom 1 through a simple two class otsu thresholding. The resulting mask is then parsed into 256*256px tissue patches at zoom 2.5. Epithelial regions appear as dark heterogeneous regions. Tissue patches undergo a gaussian smoothing so as to homogeneous epithelial regions and a final two class otsu thresholding is applied to discriminate epithelium from stroma.


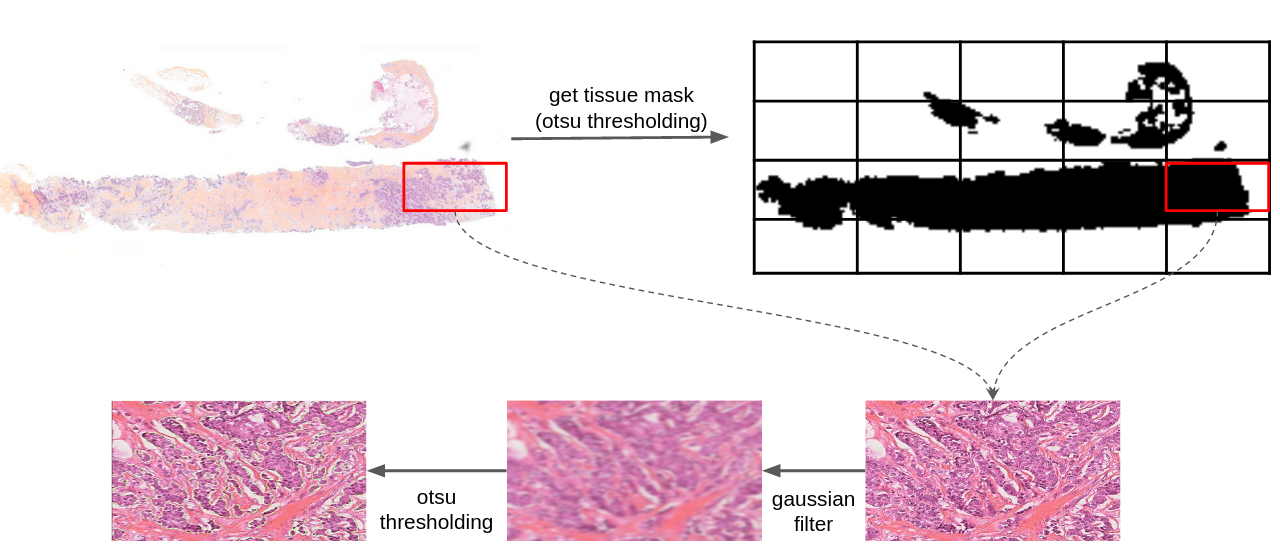


**Table A. filtering functions**

| filter | algorithm | details |
| --- | --- | --- |
| discard patches that do not contain nuclei | MobileNetV2 classifier [2] | The network was trained to distinguish patches that contain nuclei from patches that do not contain nuclei |
| blur | a laplacian filter is applied to the image. If the variance of the resulting mask is under a given threshold, then the image is filtered out | Patches that are too blurry are filtered out |
| not enough tissue | - gets the most frequent pixel value ***val*** in the image converted to greyscale- checks the proportion **prop** of pixels with intensity **i** such as \|**val** - **i**\| < **thres_1-** discards the patch if **prop** > **thres_2** | Patches that do not contain enough tissue are filtered out |

The table summarizes the various filters applied in the filtration process. Patches that do not contain nuclei, that are blurry or that contain too little tissue inside are filtered out.

**References**

1. Otsu, N. (1979). A threshold selection method from gray-level histograms. *IEEE transactions on systems, man, and cybernetics*, *9*(1), 62-66.

2. Sandler, M., Howard, A., Zhu, M., Zhmoginov, A., & Chen, L. C. (2018). Mobilenetv2: Inverted residuals and linear bottlenecks. In *Proceedings of the IEEE conference on computer vision and pattern recognition* (pp. 4510-4520).
